# Identification of Drug-Induced Liver Injury Biomarkers from Multiple Microarrays Based on Machine Learning and Bioinformatics Analysis

**DOI:** 10.3390/ijms231911945

**Published:** 2022-10-08

**Authors:** Kaiyue Wang, Lin Zhang, Lixia Li, Yi Wang, Xinqin Zhong, Chunyu Hou, Yuqi Zhang, Congying Sun, Qian Zhou, Xiaoying Wang

**Affiliations:** 1 Key Laboratory of Pharmacology of Traditional Chinese Medical Formulae, Ministry of Education, Tianjin University of Traditional Chinese Medicine, Tianjin 301617, China; 2 Key Laboratory of Component-Based Chinese Medicine, Tianjin University of Traditional Chinese Medicine, Tianjin 301617, China; 3 College of Traditional Chinese Medicine, Tianjin University of Traditional Chinese Medicine, Tianjin 301617, China

**Keywords:** drug-induced liver injury, machine learning, diagnosis, biomarker, multiple microarrays

## Abstract

Drug-induced liver injury (DILI) is the most common adverse effect of numerous drugs and a leading cause of drug withdrawal from the market. In recent years, the incidence of DILI has increased. However, diagnosing DILI remains challenging because of the lack of specific biomarkers. Hence, we used machine learning (ML) to mine multiple microarrays and identify useful genes that could contribute to diagnosing DILI. In this prospective study, we screened six eligible microarrays from the Gene Expression Omnibus (GEO) database. First, 21 differentially expressed genes (DEGs) were identified in the training set. Subsequently, a functional enrichment analysis of the DEGs was performed. We then used six ML algorithms to identify potentially useful genes. Based on receiver operating characteristic (ROC), four genes, DDIT3, GADD45A, SLC3A2, and RBM24, were identified. The average values of the area under the curve (AUC) for these four genes were higher than 0.8 in both the training and testing sets. In addition, the results of immune cell correlation analysis showed that these four genes were highly significantly correlated with multiple immune cells. Our study revealed that DDIT3, GADD45A, SLC3A2, and RBM24 could be biomarkers contributing to the identification of patients with DILI.

## 1. Introduction

Drug-induced liver injury (DILI) refers to liver damage caused by a drug itself or its metabolites (including various chemical drugs, herbal medicines, and dietary supplements) [1]. The clinical manifestations of DILI are varied, mainly including nausea, jaundice, and ascites, and the influencing factors include genetic and environmental factors. Based on the mechanism of action of the implicated drugs, DILI is classified into two types, intrinsic (e.g., acetaminophen) and idiosyncratic (e.g., non-steroidal anti-inflammatory drugs (NSAIDs) and anti-thyroid drugs) [2,3]. Intrinsic DILI is thought to be related to the drug dose, whereas idiosyncratic DILI is usually associated with the body’s immune system. Over the years, the incidence of DILI has gradually increased with the widespread use of drugs and the continuous development of new drugs, and the current global incidence is 14–19 per 100,000 [4]. Notably, a retrospective study in China reported an estimated DILI prevalence of 23.8 per 100,000 [5], which is higher than that in many Western countries, probably due to the widespread use of Chinese herbal medicines and tuberculosis drugs [6,7]. Unlike in the East, the top three drugs related to DILI in the West are antibiotics and cardiovascular and psychiatric drugs [8]. Early DILI is a reversible and controllable pathological process. Early discontinuation of the offending drug and intervention can not only block the progression of DILI but also improve its prognosis. Otherwise, DILI progresses to acute liver failure, which is extremely dangerous and possibly irreversible. In addition, DILI affects drug development and marketing, resulting in significant economic loss. Hence, DILI is a major cause of drug withdrawal from the market. However, the etiology and symptoms of DILI are complex in clinical practice, making its diagnosis challenging.

In general, clinical analysis is performed in combination with biochemical, histological, and imaging examinations. Commonly used biochemical indicators include alanine aminotransferase (ALT), aspartate aminotransferase (AST), alkaline phosphatase (ALP), and total bilirubin (TBIL) [9,10]; however, these markers are neither sensitive nor specific for DILI. Moreover, liver biopsy in patients with DILI could show a variety of histological features such as inflammation, necrosis, cholestasis, and fibrosis, which are common in many liver diseases [11]. In addition, the Roussel Uclaf Causality Assessment Method (RUACM) scale is a recommended clinical assessment tool; however, its reliability is not satisfactory enough to fully meet actual clinical needs. Currently, there is neither a specific clinical indicator for the diagnosis of DILI nor an accepted unified standard, and its diagnosis remains exclusive. Thus, physicians must carefully evaluate and exclude other causes of liver disease before making a diagnosis of DILI, which requires a high level of expertise. In particular, the clinical manifestations of idiopathic DILI are very similar to those of other acute and chronic liver diseases, but the lack of specific serological diagnostic markers has led to a lack of clinical awareness of its diagnosis. To address this challenge, there is an urgent need to identify DILI-specific biomarkers, which will help to identify DILI and thus be crucial for the prevention and timely interruption of the damaging effects of drugs on the organism to a great extent.

Increasing evidence suggests that the occurrence and development of DILI may be a multi-gene, multi-cellular, and multi-path process; therefore, it is difficult to achieve a comprehensive understanding of the pathogenesis of individual genes [12]. Given this, various high-throughput omics and gene chip technologies have played a significant role in disease diagnosis and prognosis and so far have been used in a variety of diseases, including myocardial infarction [13], Alzheimer’s disease [14], liver cancer [15], pulmonary fibrosis [16], and diabetes [17]. Genomics has also been used to explore the markers and pathogenesis of DILI [18]. Using gene chips, researchers can simultaneously detect thousands of genes’ expression; this has the advantages of rapidity, precision, and low cost. In contrast, machine learning (ML) has sophisticated algorithms that automate the organization and analysis of large-scale datasets. In recent years, MLs have been widely used in the medical field to identify markers for disease diagnosis, development, prognosis, and drug treatment, thereby helping to improve medical care. It has the advantage of detecting hard-to-identify patterns from large and/or complex data sets. ML includes various algorithms, such as least absolute shrinkage and selection operator (LASSO), support vector machine (SVM), gradient boosting machine (GBM), random forests (RF), decision trees (DT), and neural networks (NN) chosen for this work.

LASSO, a typical ML technique, is commonly used for the prediction of various diseases, especially tumor diagnosis. Y. Zhou used Lasso to develop the radiologic features based on dynamic contrast-enhanced (DCE) MR images to predict the microvascular invasion (MVI) in mass-forming intrahepatic cholangiocarcinoma (IMCC) before surgery. The model showed good accuracy, sensitivity, and reproducibility [19]. SVM, a very popular machine learning algorithm with maximum (support) separation bounds (vectors), classifies data based on its features. Compared to other ML methods, SVM is very powerful in identifying subtle patterns in complex datasets [20] and has been used for many years in various disease-classification analyses [21]. In addition, SVM can be used to predict potential drug targets. Some studies evaluated the performance of SVM in identifying drug targets in hepatocellular carcinoma, and the mean AUC score for 10 repetitions was 0.8834, and the worst was 0.8820, which indicates that SVM has relatively stable predictive performance [22]. GBM is also a widely used machine learning technique that uses the XGBoost package to generate powerful predictive models through the integration of multiple weak models (e.g., DT) and is a well-established technique for solving regression and classification problems [23]. Five machine algorithms, GBM, SVM, DT, RF, and LR logistic regression, were systematically compared to predict NAFLD, NASH, and advanced fibrosis and found that GBM performed best, followed by RF, with AUCs all greater than 0.80 [24]. DT and RF are two tree-based machine learning methods that are widely used for data mining and disease prediction. Decision trees have a good history of supporting diagnosis in the medical field, using categorical and numerical data with the aim of assigning samples to specific classes [25]. In fact, due to the transparency of the decision rules determined by the algorithm [26], this method is particularly suitable for diagnosis. For example, a study has already demonstrated serum zinc as a predictor for identifying individuals with vitamin D deficiency through the use of decision trees [27]. In order to improve the accuracy of DT prediction, Leo Breiman proposed RF in 2001 [28]. RF is a model consisting of a number of random decision trees that give their own predictions to determine the best solution with the highest number of votes as the final decision [29]. The high precision used to predict diseases has been confirmed in previous reports. D. Sharma compared seven different machine learning methods to identify NAFLD and CVD and finally proposed that the RF model had the best performance, followed by Lasso [30]. Yen JS studied and developed random forest and logistics regression models to predict acetaminophen-induced hepatotoxicity and found that the random forest model results (AUC = 0.98) were superior to roentgen regression (AUC = 0.68) [31]. NN mimic the potential learning capabilities of the human brain and thus belong to the field of artificial intelligence, which is modeled as a series of neurons (or nodes) organized in layers, where each neuron in one layer is connected to neurons in other layers with associated weights, thus minimizing errors in the learning task [25,32]. F. Hammann [33] proposed an integrated model (decision tree, K-nearest neighbor, support vector machine, neural network) for clinically relevant DILI prediction based on drug structure alone, achieving a corrected classification rate of 89%. In addition, to achieve better clinician triage decisions, Y. Raita developed four ML models, Lasso, RF, gradient augmented decision tree, and deep neural network, and found that all models performed well in predicting critical care and hospitalization outcomes [34].

There have been many studies on MLs, and different algorithms have different levels; therefore, combining multiple ML techniques will greatly improve the credibility of the analysis results.

Several laboratory and clinical studies have revealed that there are differences in transcriptomes between patients with DILI and healthy individuals. In our study, we collected multiple DILI microarrays from the Gene Expression Omnibus (GEO) dataset to identify biomarkers for DILI diagnosis using six ML algorithms. The results were confirmed by differential and area under the curve (AUC) analysis in the testing groups. It is worth mentioning that, to our knowledge, this may be the first study to use ML to mine genes from the GEO database that are useful for DILI diagnosis, hoping to provide a scientific basis for future clinical diagnosis.

## 2. Results

### 2.1. GEO Dataset Preparation

**Figure 1:**
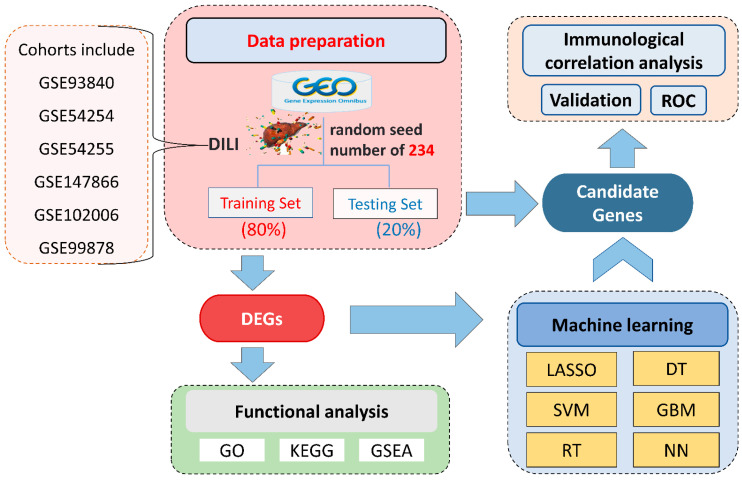
Illustration of the overall workflow. DILI, drug-induced liver injury; GEO, Gene Expression Omnibus; DEGs, differentially expressed genes; GO, gene ontology; KEGG, Kyoto Encyclopedia of Genes and Genomes; GSEA, gene-set enrichment analysis; SVM, support vector machine; LASSO, least absolute shrinkage and selection operator; RT, random forest; GBM, gradient boosting machine; DT, decision tree; NN, neural network; ROC, receiver operating characteristic.

Based on the inclusion and exclusion criteria, six microarrays (GSE93840, GSE54254, GSE54255, GSE147866, is shown in Figure 1. Note that not all samples in the microarray were included, and Supplementary Table S1 summarizes the dataset information, including 103 DILI samples and 29 control samples. Using the random seed method, with 234 as the random seed number, we divided it into a training set with 105 samples (including 84 DILI and 21 healthy samples) and a testing set containing 27 samples (19 DILI and 8 healthy) based on a ratio of 80:20 [35,36,37].

### 2.2. Identification of DEGs

**Figure 2:**
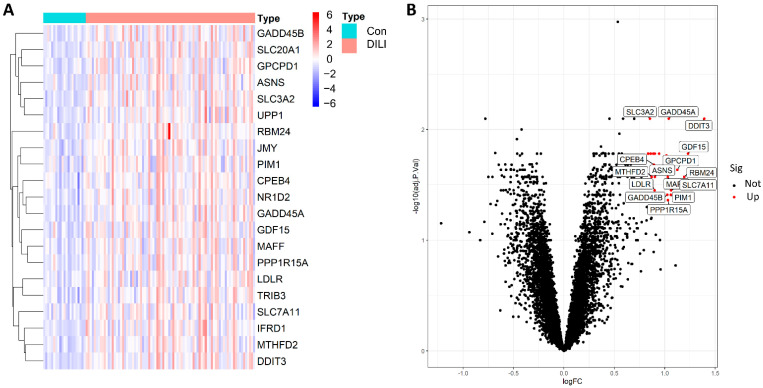
The 21 DEGs were distributed in both the DILI group and the control group (|log FC| > 0.8, FDR < 0.05, and *p*-value < 0.05). (**A**): heatmap of 21 DEGs between the Con and DILI groups; (**B**), volcano diagram of all genes; red represents significantly upregulated genes in the DILI group compared to Con group, and black represents not significantly upregulated genes. R software (version 4.1.4; written by Ross Ihaka and Robert Gentleman from the University of Auckland originally, N.z., and maintained by Lucent Technologies, USA, https://www.r-project.org/) was used to create the maps, including R package pheatmap (version 1.0.12; created by Raivo Kolde’ team; https://cran.r-project.org/web/packages/pheatmap/index.html) for the heatmap and ggplot2 (version 3.35; written by Hadley Wickham; RStudio, USA, https://cran.r-project.org/web/packages/ggplot2/index.html) for the volcano plot, respectively. FC, fold change; FDR, false-discovery rate.

In the training set, we identified 21 biologically significant differentially expressed genes (DEGs), as detailed in Supplementary Table S2. A volcano plot showed that, compared with the control samples, all 21 DEGs were significantly upregulated in the DILI samples. Generally speaking, the larger the absolute value of Log FC and adjusted P value of −Log10, the greater the difference between the two groups. Therefore, growth arrest and DNA damage-inducible protein GADD45 α (GADD45A) and DNA damage-inducible transcript 3 protein (DDIT3) were distributed to the edges of the heatmap, indicating a clear difference between the two groups (Figure 2).

### 2.3. Functional and Pathway Enrichment Analysis

**Figure 3:**
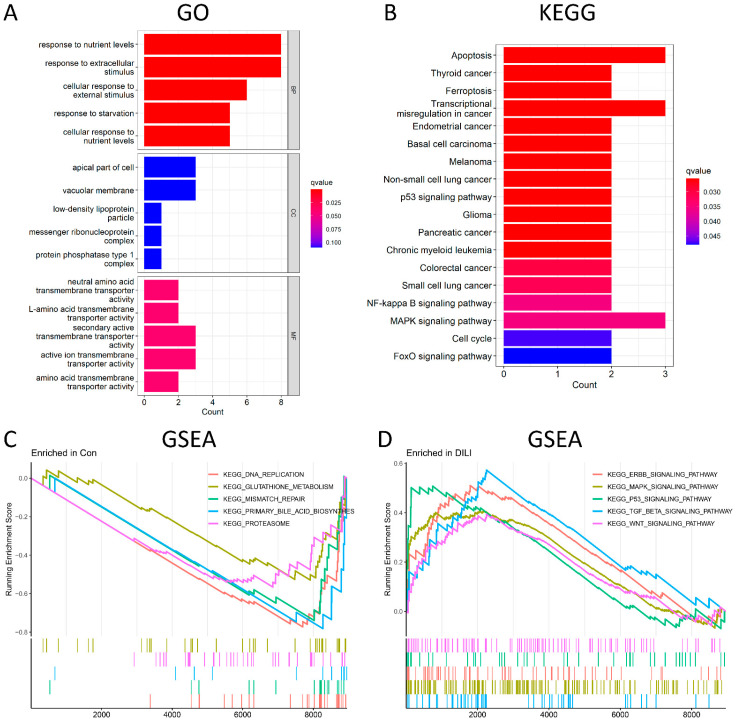
Functional and pathway enrichment analysis. (**A**): The top−5 BP terms, top−5 CC terms, and top−5 MF terms enriched in GO term. (**B**) The 18 significantly enriched KEGG pathways. (**C**) The top−5 most significantly enriched GSEA-KEGG control terms. (**D**) The top−5 most significantly enriched GSEA-KEGG DILI terms. BP, biological process; CC, cellular component; MF, molecular function. Count represents the number of genes enriched in the GO or KEGG entry. The Q value is the *p* value after multiple correction, which is represented by color. The redder it is, the smaller the q value is and the more obvious the enrichment is.

GO analysis was conducted from three different aspects, namely, biological process (BP), cellular component (CC), and molecular function (MF). According to the GO enrichment results (Figure 3A and Supplementary Table S3), 94 terms were enriched in 21 DEGs. The top-5 enriched BP terms include response to nutrient levels, response to extracellular stimulus, cellular response to external stimulus, response to starvation, and cellular response to nutrient levels. Among them, response to nutrient levels was significantly activated, with 8 upregulated genes involved, including DDIT3, UPP1, GDF15, JMY, CPEB4, ASNS, LDLR, and PIM1. However, the top-5 enriched CC terms were not significant. The most enriched MF term was neutral amino acid transmembrane transporter activity. Among the KEGG pathways (Figure 3B), the top five were significantly enriched in apoptotic, tumor-related, and ferroptosis signaling pathways. Among them, apoptosis was the most significantly activated, with 3 upregulated genes involved, including DDIT3, GADD45A, and GADD45B. To further investigate the pathway analysis of DEGs, GSEA analysis was performed on control and DILI groups, respectively. In the control group (Figure 3C), GSEA was significantly enriched in DNA replication, glutathione metabolism, mismatch repair, primary bile acid biosynthesis, and proteasomes. Meanwhile, GESA in the DILI group (Figure 3D) showed significant enrichment in the ERBB signaling pathway, MAPK signaling pathway, P53 signaling pathway, TGF-β signaling pathway, and WNT signaling pathways.

### 2.4. Six ML Algorithms Developed for Diagnostic Models

**Figure 4:**
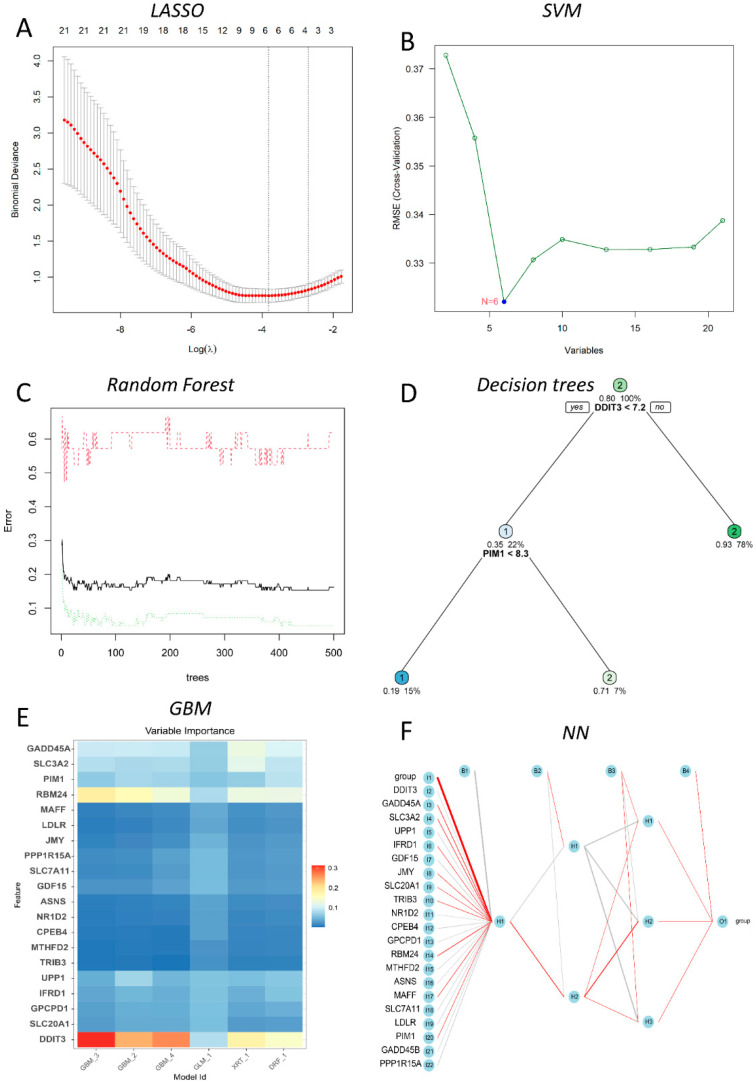
Six ML algorithms developed for DILI diagnosis. (**A**) LASSO for 4 prognostic DEGs (the bottom and top abscissa shows the Log(λ) value and number of variables; the ordinate show the binomial deviance); (**B**) SVM for 21 prognostic DGEs (the abscissa shows the number of variables, and the ordinate shows the root mean square error); (**C**) RF for the classification of control and DILI individuals (the abscissa shows the error change with the number of the ordinate trees); (**D**) the optimal decision trees for the classification of control and DILI individuals with the gene-expression values and allocated probability; (**E**) six-fold in GBM for the classification of control and DILI individuals (the variable importance for each gene of multiple GMB models); (**F**) NN for the classification of control and DILI individuals with three hidden layers.

**Figure 5:**
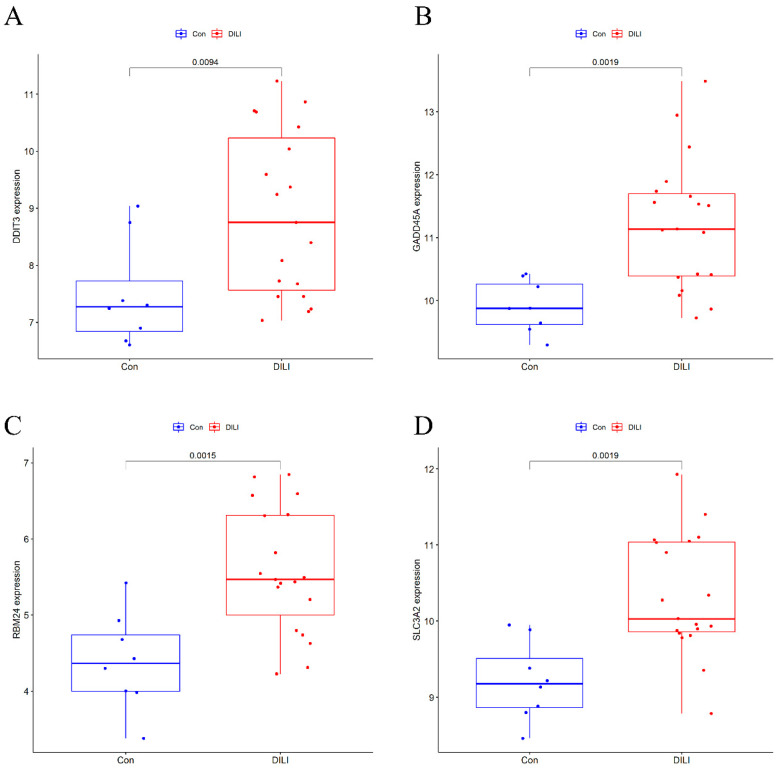
Results of a comparison of 4 candidate DEGs in testing set. Compared with Con, *p* < 0.05 as statistic difference. (**A**) The expression of DDIT3 between the Control and DILI groups in the testing set; (**B**) The expression of GADD45A between the Control and DILI groups in the testing set; (**C**) The expression of RBM24 between the Control and DILI groups in the testing set; (**D**) The expression of SLC3A2 between the Control and DILI groups in the testing set.

In this study, six prediction models—LASSO, SVM, DT, RF, NN, and GBM—were successfully built (Figure 4), and the error rates of six MLs are shown in Table 1. Using the LASSO algorithm (Figure 4A), four optimal genes—DDIT3, GADD45A, 4F2 cell-surface antigen heavy chain (SLC3A2), and RNA-binding protein 24 (RBM24)—were filtered out. The error rates of the training and testing sets were 3.8% and 29.6%, respectively (Table 1). Six candidate genes were identified using SVM (Figure 4B), with 2 costs and 37 support vectors for minimal root mean square error (RMSE), and the error rates of the training and testing sets were 3.8% and 7.4%, respectively (Table 1). In RF (Figure 4C), the number of variables tried at each split was 4 with 500 trees, and the error rates of the training and testing sets were 1.9% and 0% respectively (Table 1). In DT (Figure 4D), the optimal trees showed that the 7.2 threshold of DDIT3 might be conducive to distinguishing health from DILI, and the error rates of the training and testing sets were 9.5% and 14.8%, respectively (Table 1). In GBM (Figure 4E), four genes—DDIT3, GADD45A, SLC3A2, and RBM24—were predominant among the various important genes. Among them, DDIT3 and RBM24 were weighted more than the other candidate genes, and the error rates of the training and testing sets were 14.0% and 14.8%, respectively (Table 1). With the last ML, NN (Figure 4F), we found that three hidden layers could distinguish health from DILI and that the error rates of the training and testing sets were 5.0% and 11.1%, respectively (Table 1). Among these models, we filtered the most important genes according to primary weights (Supplementary Table S6). Furthermore, we normalized the weights by dividing them by the absolute values of max weights (Table 2). Four genes (DDIT3, GADD45A, SLC3A2, and RBM24) showed overall weights > 1. The above MLs optimize four candidates’ genes for the diagnosis of DILI and control. Next, we validated the four genes in the testing set, and the results showed that they were all statistically significant (*p* < 0.05) (Figure 5).

### 2.5. Evaluation of Diagnostic Value

**Figure 6:**
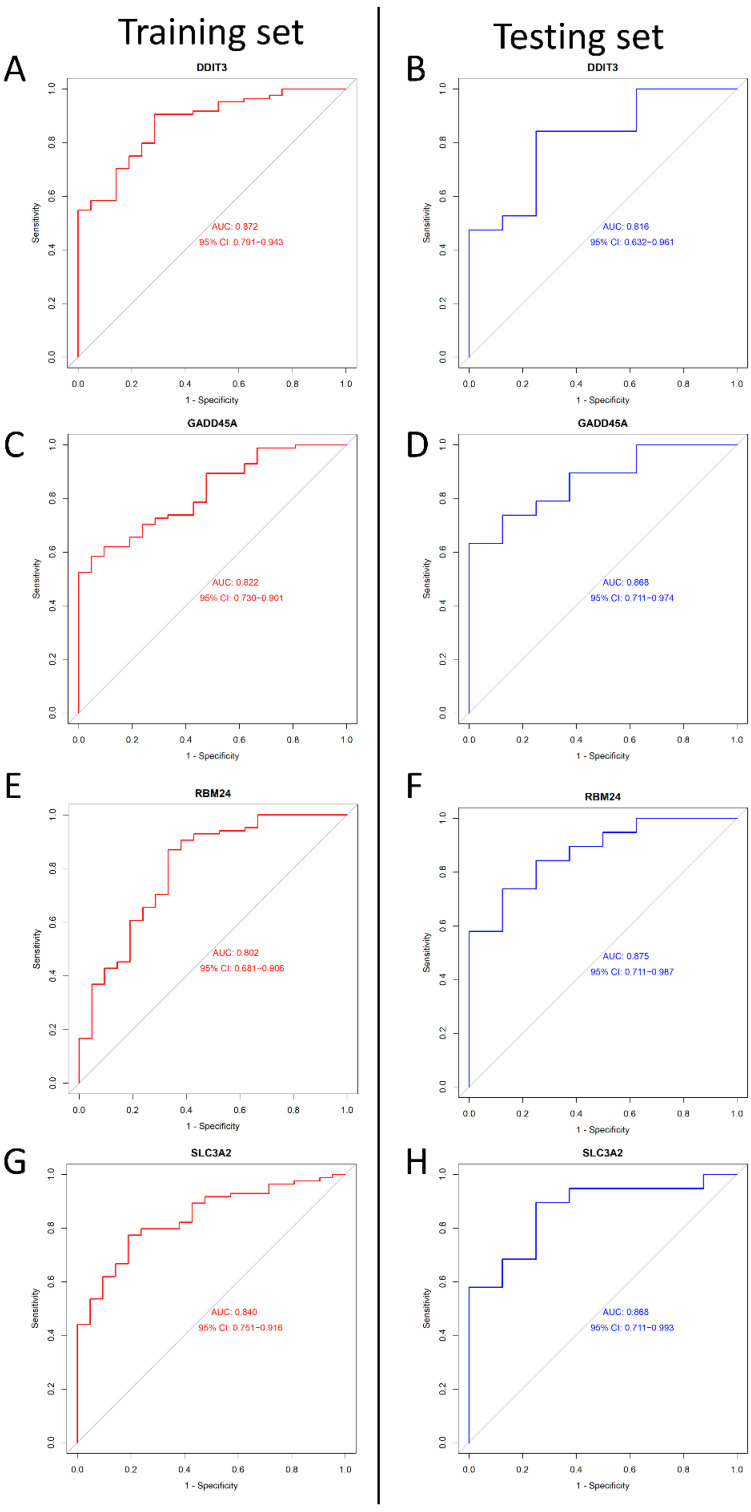
The ROC curves of DDIT3, DDIT3, SLC3A2, and RBM24 between the training and testing groups. (**A**) The ROC curve of DDIT3 in the training group. (**B**) The ROC curve of DDIT3 in the testing group. (**C**) The ROC curve of GADD45A in the training group. (**D**) The ROC curve of GADD45A in the testing group. (**E**) The ROC curve of RBM24 in training group. (**F**) The ROC curve of RBM24 in the testing group. (**G**) The ROC curve of SLC3A2 in training group. (**H**) The ROC curve of SLC3A2 in the testing group. Red represents training, and blue represents testing.

We adopted the ROC curve and AUC values to assess the diagnostic value of the four genes. When we set the four genes (as mentioned above) into the ROC curve, the results showed that their AUC values were >0.8 in both the training and testing sets (Figure 6). In conclusion, DDIT3, GADD45A, SLC3A2, and RBM24 may be diagnostic genes for DILI.

### 2.6. Immunological Correlation Analysis

**Figure 7:**
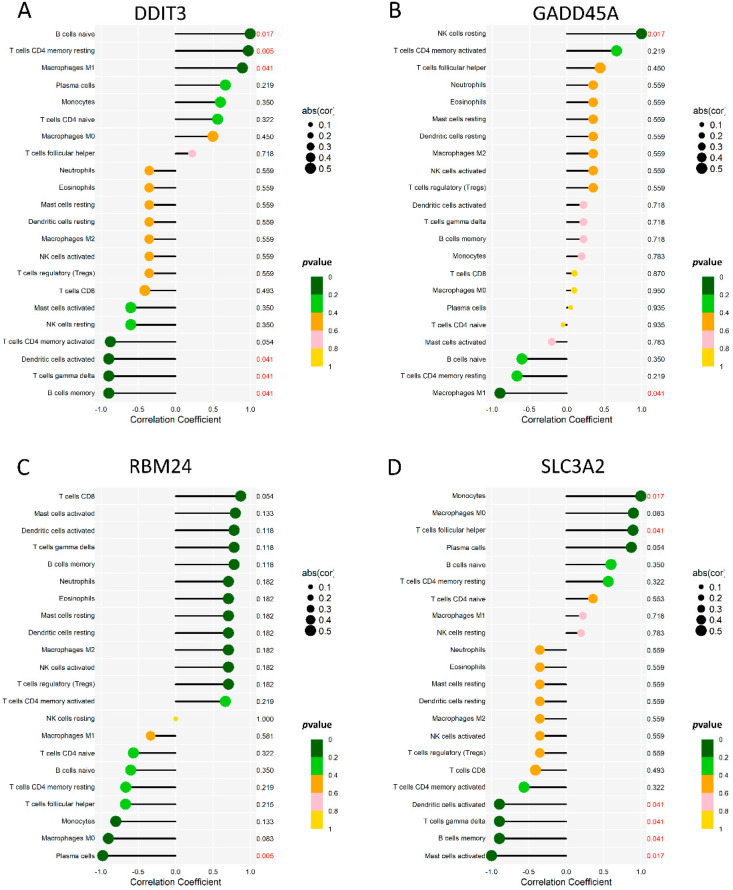
The immune correlation between 4 genes and 22 immune cells. (**A**) A lollipop map of DDIT3 and 22 immune cells. (**B**) A lollipop map of GADD45A and 22 immune cells. (**C**) A lollipop map of RBM24 and 22 immune cells. (**D**) A lollipop map of SLC3A2 and 22 immune cells.

To analyze the correlation between the diagnostic genes and 22 immune cells, we demonstrated the immune cells’ correlation among 132 samples of six microarrays (Supplementary Figure S1). Moreover, we used Spearman’s correlation analysis to evaluate the correlations among 22 immune cells and the four diagnostic genes (Figure 7). DDIT3 (Figure 7A) showed significant correlations with B cells memory, B cells naive, dendritic cells activated, macrophages M1, T cells CD4 memory resting, and T cells gamma delta. GADD45A (Figure 7B) showed significant correlations with the NK cells resting and macrophages M1 showed significant correlations. RBM24 (Figure 7C) showed significant correlations with the plasma cells. SLC3A2 (Figure 7D) showed significant correlations with the B cells memory, dendritic cells activated, mast cells activated, monocytes, T cells follicular helper, and T cells gamma delta showed significant correlations. Linear regression maps of these four genes correlated with their respective significant immune cells are shown in Supplementary Figure S2.

## 3. Discussion

To our knowledge, this is the first study to identify biomarkers for DILI diagnosis based on information from the GEO dataset. Although DILI has become common in clinical practice in recent years, its diagnosis and differentiation from other diseases remain unclear. Previous studies have suggested that different kinds of drugs could cause liver injury, and DILI covers almost all known types of liver injury, simplified into hepatocellular, cholestatic, and mixed injuries [38]. However, in the same way, they all eventually lead to acute liver failure, with subsequent consequences of liver transplantation or death. Therefore, DILI is a health problem that cannot be ignored. Interestingly, not all drugs that cause abnormal liver test results lead to persistent liver injury, and in some cases, the continued ingestion of the suspected drugs (e.g., isoniazid) can lead to a normalization of transaminase levels [39]. Furthermore, previous studies have revealed that elevated serum transaminase levels were observed in healthy individuals consuming a high-carbohydrate, high-calorie diet [40]. Recent studies have revealed that serum ALT and TBIL levels are significantly higher in heavy drinkers than non-drinkers [41,42]. Taken together, dietary habits and alcohol intake affect transaminase levels in healthy individuals, suggesting that existing biochemical indicators for the diagnosis of DILI lack specificity. Previous studies have tried to identify biomarkers specific for DILI, including promising microRNAs, cytokeratin-18, and high-mobility-group box-1 protein [43]; however, these studies only focused on single or multiple genes, so the conclusions were still limited. To date, there is no single diagnostic tool or marker specific for DILI.

The pathogenesis of DILI is complex and unclear, and its occurrence and development do not result from a single pathway. Currently, the “three-step mechanism” is the most widely recognized, includes initial injury, mitochondrial permeability transition, and apoptosis or necrosis [44]. Currently, ML models show superior performance in disease diagnosis compared to traditional methods. Performance is the most critical for ML, and accurate analysis under high performance is the ultimate goal of building ML models. Supriya et al. [45] summarized the common performance metrics of current ML models, including accuracy, calibration, discrimination, negative predictive value, positive predictive value, recall, and specificity. Based on the performance above, compared to traditional methods, the advantages of ML is mainly reflected in its flexibility and scalability. ML is a sub-discipline of artificial intelligence wherein learning algorithms can be developed based on a series of complex algorithmic processes used to organize and analyze large data sets, ultimately aiding in good decision-makings and predictions [46]. The application of ML models could enhance the accuracy of clinical diagnosis, leading to better treatment. The present study has good prospects because no published studies have mined the GEO database to identify diagnostic markers specific for DILI. Second, we developed six ML algorithms and performed weighting to improve the reliability of the analysis results. The GEO database, an international publicly accessible data repository, contains interesting datasets and visualizes and analyzes data, greatly reducing research costs and improving research efficiency [47]. In the present study, we retrieved six datasets from the GEO database according to our inclusion and exclusion criteria and analyzed transcriptomic data between healthy and DILI samples, including 19 drugs, mainly non-steroidal anti-inflammatory drugs and antibacterial drugs, both of which are main causes of DILI. Given the large number of drugs that cause DILI, recent studies on identifying their common markers have been less promising. Based on this, we hope to identify their common genes by ML to provide a basis for identifying DILI. In this study, we set the random seed at 234 with the *set.seed* function to separate the training and testing sets, and the microarrays were randomly divided into the training and testing sets in a ratio of 8:2. In fact, we found that the segmentation of the training and testing data has different division standards in the different articles, such as 90:10 [48], 85:15 [49], 80:20, 70:30 [50], 60:40 [51], and so on. We choose 8:2 for three reasons: first, it is supported by the reported literature; second, considering that the number of samples is not large enough, we wanted to make the training data for developing the model as large as possible; third, the ratio of the number of DILI samples and control samples is about 80:20. In the training set, we found 21 DEGs, and the reason for this small number may be the difference in the molecular mechanisms by which different drugs cause liver injury. These 21 DEGs are common to the different drugs. Functional enrichment analysis was performed on the 21 DEGs. Results showed that these genes were significantly enriched in the cellular response to external stimulus, apoptosis, and ferroptosis signaling pathways, which are known to play a central role in the development of liver damage.

Subsequently, we developed and optimized six ML algorithms and successfully established a DILI diagnostic prediction model. It was worth noting that, in this work, we paid more attention to the values of the weights rather than the models, although we established six models of ML for classification. Therefore, we are not exaggerating to say that our method is the best but also that we can extract useful information about DILI diagnosis well in these six algorithms. For the selection of these six algorithms, the main considerations were the algorithm’s matching to the sample data, a wide range of applications in disease classification and diagnosis, and prediction performance. LASSO is one of the most commonly used algorithms, and its prediction performance has been demonstrated in previous studies [52]. In this study, the four most important genes screened in Lasso also have relatively high weights in other MLs, and 17 genes with zero weight in LASSO also have good weights in other MLs. Therefore, we compared the weights of all genes with or without Lasso analysis separately and concluded that, although the Lasso filtering ability was aggressive, it did not affect our final results. In addition, for nonlinear and complex relationships of high-dimensional variables, SVM is usually more effective than common statistical methods, such as Logistic regression (LR), and especially SVM shows more advantages in small-sample data analysis, so we chose SVM rather than LR to predict DILI. In our study, data from the control and DILI groups were generated to build the SVM model. We used a portion of the data to build the training set, and the model constructed a hyperplane to divide it into two different classes. The DILI was located on one side of the hyperplane, and the other points were classified as control [53,54]. Then, we built a testing set of data to validate it with the rest of the toxic data mentioned above, and the results were more satisfactory. RF is a typical and highly sophisticated ML, and its ability to predict disease has been demonstrated in earlier studies; for example, the Wu CC developed four models to predict fatty liver, and the ROC results showed that the RF model showed higher performance than several other models, with an ROC of 0.925 for RF [55]. In addition, we used DT to divide the training set data into different groups according to the features of DDIT3 (DDIT3 > 0.72 or DDIT3 < 0.72) and then split them again according to PIMI to generate smaller groups. However, it is important to note here that fitting DT that contains all data for a single class can lead to overfitting.In other words, since the DT classifies only the training set samples and not the total samples, the accuracy for the total is likely to be much lower than that for the training samples in most cases [54]. GBM builds models by combining information from multiple DTs in a step-by-step manner [56], a process that is repeated hundreds or thousands of times. That is, the computer uses augmentation and integration to learn multiple simple algorithms to create a larger pool of algorithms, resulting in higher predictive performance. The main advantage of this method is that it combines the interactions between variables, thus avoiding the influence of extreme values and is less prone to overfitting [23,57]. This was confirmed by the fact that the results of our testing set were similar to those of the training set. However, GBM is not without its shortcomings. The biggest problem is memory consumption and the accompanying slow calculation speed [58]. Of course, if the sample size is large enough, other algorithms will also have these drawbacks. NNs have been developed for widespread use in disease diagnosis, and they can be very valuable in improving prediction accuracy, especially when the model form and the relationship between variables may be non-linear or unknown [25]. It is worth mentioning that an important difference between NNs and other ML techniques is that NNs can learn by themselves which features in the training data are important for classification or prediction tasks, rather than being told by domain experts which features to use [59]. Based on continuous exploration by the research team, six algorithms were optimized based on weights to screen the more accurate useful genes, and four genes that are helpful for DILI diagnosis were finally identified: DDIT3, GADD45A, RBM24, and SLC3A2. ROC curve analysis was a standard method to evaluate performance, and AUC was used by us to measure the predictive performance of the model. It was found that the AUC values of these four genes in the training and testing sets were all greater than 0.80, showing greater stability and higher values than previous ML models [60]. It is known from the literature that these four genes have been widely reported in other diseases, especially tumors, consistent with our KEGG and GSEA analyses results; however, few studies have focused on their relationship with DILI.

As the name implies, DDIT3, also known as C/EBP homologous protein (CHOP), is a key stress-responsive transcription factor that is activated in a variety of cellular stress responses, including DNA damage, endoplasmic reticulum stress, hypoxia, and amino acid starvation, thereby inducing cell cycle arrest and apoptosis. The KEGG analyses results showed that these genes were mainly enriched in apoptosis, ferroptosis, and various tumor-related pathways. Indeed, apoptosis and ferroptosis are also inseparable from tumor development; unlike apoptosis, ferroptosis is an iron-overload necrosis pathway that occurs due to the inhibition of cystine–glutamate exchangers on the cytoplasmic membrane and is characterized by the accumulation of lipid peroxides [61,62]. Previous studies have shown that ferroptosis promotes the expression of ATF-dependent genes, including CHOP, tribbles homolog 3 (TRIB3), and asparagine synthetase [glutamine-hydrolyzing] (ASNS) [63]. In addition, it has been shown that tumor growth is heavily dependent on glutamine and that glutamine deprivation increases DDIT3 expression through activating transcription factor-4 (ATF4)-mediated transcription [64]. In particular, DDIT3 shows strong high expression in myxoid liposarcoma tissues. The genetic location of the myxoid liposarcoma is a repeated translocation of DDIT3 on chromosome 12, which produces a chimeric oncoprotein [65]. In addition to its role in tumors, DDIT3 is also reportedly induced by various cytotoxic drugs and participates in the development of hepatotoxicity [66,67]. A previous study demonstrated that cell damage initiated by diclofenac and carbamazepine was directly related to the expression of CHOP [68] and that inhibiting CHOP prevented diclofenac/tumor necrosis factor α (TNFα)-mediated apoptosis. The key event occurring in hepatotoxicity is hepatocyte deaths, which is closely related to endoplasmic reticulum stress response. The transcriptome results of a large number of DILI compounds in primary hepatocytes confirmed the activation of the ATF4-CHOP pathway [69], which was consistent with the results of our analysis. Furthermore, in hepatotoxic drug-treated mice, DDIT3 was found to be activated by the unfolded protein response (UPR) via the PERK pathway [70]; that is, DDIT3 acted as a transcription center to maintain endoplasmic reticulum protein stability [71]. Therefore, we suspect that DDIT3 may be a marker for predicting DILI in the future.

GADD45A is a stress gene subject to conditioning by P53, which is involved in biological functions such as DNA damage repair, cell cycle arrest, apoptosis, and tumor inhibition. Recent reports have suggested that decreased GADD45A expression due to abnormal methylation may contribute to cancer cell resistance to radiotherapy through the PI3K/AKT signaling pathway [72,73]. Moreover, studies have shown that the upregulation of GADD45A inhibits the bladder cancer cell cycle and is regulated by P53 [74]. In addition to tumor studies, other studies found that tetrachloromethane (CCl_4_)-induced hepatic fibrosis in mice was accompanied by the downregulation of GADD45A and the upregulation of transforming growth factor (TGF-β)/Smad, ultimately confirming that GADD45A regulates the activation of hepatic stellate cells by inhibiting TGF-β/Smad signaling, thereby preventing liver fibrosis [75].

RBM24 contains a conserved RNA recognition motif (RBM) composed of RNP1 and RNP2 subunits [76]. Like GADD45A, RBM24 is also a downstream target of P53. It has been demonstrated that RBM24 is an essential gene in cardiovascular development and sarcomere assembly [77,78]. Some years ago, RBM24 was identified as a key splicing factor in cardiac development, and data have revealed that RBM24-deficient mice died during embryonic development, partly due to the abnormal activation of p53-dependent apoptosis [79]. Conversely, AAV9-mediated RBM24 overexpression reportedly led to cardiac fibrosis in adult mice, possibly by regulating the TGFβ signaling pathway [80]. As a tumor suppressor, abnormally high levels of RBM24 expression, induced by drugs, can lead to hepatocyte death. As mentioned above, hepatocyte death is critical for DILI; therefore, we consider RBM24 a marker for the diagnosis of DILI.

It is well known that enzymes play an important role in the metabolism of drugs after entering the body, but in fact, another class of molecules—transporter proteins, especially solute carriers (SLC)—also play an indispensable role. Of note, SLC3A2, an important member of the SLC family, plays a role in regulating the transmembrane transport of amino acids. In this study, we found that the AUC of the SLC3A2 gene in both the training and testing sets was above 0.80, implying that it could be used as a marker for the diagnosis of DILI. Interestingly, no study has reported its association with DILI. However, many studies have shown that SLC3A2 is highly expressed in most tumor types [81]. Recent studies have shown that the IFN γ released by CD8+ T cells downregulates the expression of SLC3A2 and SLC7A11 and impairs the uptake of cystine by tumor cells, thereby promoting lipid peroxidation and ferroptosis in tumor cells [82]. In addition, studies have confirmed that SLC overexpression activates AKT and its downstream signaling pathway [83] and regulates tumor cell proliferation and survival, apoptosis, and autophagy to promote tumor development. In fact, we are well aware that the diagnosis of tumors in clinical practice mainly relies on biopsy and that the clinical manifestations of DILI are very different from those of tumors. Therefore, we recommend the use of SLC3A2 for the diagnosis of DILI.

We are well aware that the present work, although innovative and interesting, has certain limitations. Firstly, this is a retrospective case-control study, which is more susceptible to selection bias than other epidemiological studies. Secondly, an inadequate sample number is a common limitation of bioinformatics research, although we have validated the prediction model on a test set based on AUC values. Thirdly, our current level of machine learning is rudimentary, and the detection performance metrics are relatively single. Lastly, it is well known that the “black box” characteristics of machine learning algorithms (especially NNs) may be difficult to explain. In the future, it is necessary to further verify these results through in vitro experiments. However, we still hope that our current work can be of some use to relevant research and clinical diagnosis.

## 4. Materials and Methods

### 4.1. GEO Database Download and Data Preparation

We searched the GEO database with “drug-induced liver injury” as the keyword. The dataset was screened according to the following criteria: (1) the biological species was human; (2) the item type was series ID; (3) the study type was expression profiling by array; (4) expression profiles included both healthy samples and DILI samples. The exclusion criteria were as follows: (1) presence of viral hepatitis or other liver diseases and (2) samples with gene knockout.

### 4.2. Data Processing

First, the *sva* R package (version 3.42, Biocounder, USA) and *preprocessCore* (version 1.56.0, Biocounder, USA) were applied to eliminate branch effects and quantile normalization among the multiple microarrays that met the inclusion criteria. Subsequently, we set the random seed at 234 with the *set seed* function to separate the training and testing sets in a ratio of 80:20. The training set was used to develop the prediction model, and, as its name implies, the testing set was used to validate the results of the model.

### 4.3. Identification of DILI-Related DEGs

We used the *limma R* package (version 3.52.4, created by Gordon Smyth, Biocounder, USA) to screen the DEGs in of both the DILI and control samples in the training set. After quantile normalization, the raw data were log2 transformed. According to the method of Benjamini and Hochberg, the *p*-value was adjusted to control for the FDR. We filtered DEGs based on the following criteria: |logFC | > 0.8 [84], FDR < 0.05 (Student’s *t*-test), and *p*-value < 0.05.

### 4.4. Functional and Pathway Enrichment Analysis

The DEGs obtained by the above methods were subjected to functional and pathway enrichment analysis, specifically including GO, KEGG pathway analysis, and GSEA based on the *clusterProfiler*, *DOSE*, and *enrichplot* packages of R, versions 3.16.1, 3.14.0, and 1.8.1, respectively. GO analysis was composed of MF, BP, and CC.

### 4.5. MLs Developed for Diagnostic Models

With the above DEGs, we built adopted six ML algorithms (LASSO, SVM, DT, RF, GBM and NN) to classify the DILI and healthy control. Furthermore, the error rate was adopted to verify the accuracy in both training and testing sets. The LASSO algorithm was adopted by the *glmnet* (version 4.1–4, created by Trevor Hastie’ team) R package. In this R package, the function *cv.glmnet* was adopted to tune the value of lambda. Then, the R package *glmnet* was adopted to the model. Furthermore, we set the min lambda as 2000, family as “binomial”, ten folds, and type of measure as “class”. With the tuned parameter, we built an optimal LASSO model for the validation of the testing set. The SVM algorithm was accomplished by the *e1071* R package (version 1.7–9, created by David Meyer’ team). In *e1071*, function *tune.svm* was added to tune the parameter. We set the kernel as “linear”; the cost was from 1 to 20, with a step size of one, and ten folds. With the tuned parameter, we built an optimal SVM model for the validation of the testing set. The DT algorithm was accomplished by the rpart R package (version 4.1.16, created by Terry Therneau’ team) and rpart.plot R package (version 3.1.1, created by Stephen Milborrow’ team). In this package, the rpart function was served to the model; the method was set as “class” and the cp value as 0.000001, with ten folds. With the DT model, we used the testing set for validation. The RF algorithm was accomplished by the randomForest R package (version 4.6–14, created by Andy Liaw’ team). In this package, the tuneRF function was served to tune the parameter, with a step factor of 1, 500 trees, and ten folds. With the tuned parameter, we built an optimal RF model for the validation of the testing set. The NN algorithm was accomplished by the *neuralnet* R package (version 1.44.2, created by Stefan Fritsch’ team). In this package, the *neuralnet* function was served to the model, with 3 hidden layers, with *err.fct* as “sse”, linear output, and ten folds. With the RF model, we used the testing set for validation. Different from the other algorithms, the GBM algorithm uses more processes and is error-prone. The *GMB* algorithm was accomplished by the *h2o* R package (version 3.36.0.3, created by Erin LeDell’ team). Because the *h2o* needs the JAVA operating environment. We downloaded and installed jdk (version1.8.0_341, Oracle Corporation, USA); jdk is free and public (https://www.oracle.com/java/technologies/downloads/, accessed on 10 August 2022). Firstly, the *h2o.init* function served to adjust the running memory (we set it with 8G). Different from the above other algorithms, both the training and the testing sets must be transformed into the h2o format with the *as.h2o* function. Then, the function *h2o.gbm* was served to the model and to tune the parameters, setting the distribution as “bernoulli”, with 100 trees, a learn rate of 0.01, a sample rate of 0.8, and ten folds. With the optimal GBM model, we took the testing set for validation. With the above process, each MLs harvests the variable weight of DEGs. Importantly, we normalized and summed the absolute values of the various weights of the DEGs using the formula: $Overall\;weights=\frac{\;abs\left(LASSO\right)}{abs\left(LASSOmax\right)}+\frac{abs\left(SVM\right)}{abs\left(SVMmax\right)}+\frac{abs\left(DT\right)}{abs\left(DTmax\right)}+\frac{abs\left(RT\right)}{abs\left(RTmax\right)}+\frac{abs\left(GBM\right)}{abs\left(GBMmax\right)}+\frac{abs\left(NN\right)}{abs\left(NNmax\right)}$. With the overall weights of six MLs, we screened the final diagnostic genes with an overall weight of >3 and further verified the expression level of candidate genes in the test set. To estimate the predicted value for DILI diagnosis, *pROC* package (Version 1.17.0.1, created by Xavier Robin’ team) was used to obtained the ROC. The AUC was calculated to determine the diagnostic value of the healthy and DILI samples. The higher the AUC value, the higher the diagnostic value.

### 4.6. Immunological Correlation Analysis

In order to analyze the correlation between useful genes and immune cells, Spearman’s rank correlation was used to analyze the correlation between 22 immune cells and four diagnostic genes, DDIT3, GADD45A, SLC3A2, and RBM24.

### 4.7. Statistical Analysis

Statistical analysis was performed with R software (version 4.1.4, written by Ross Ihaka and Robert Gentleman from the University of Auckland originally, N.z., and maintained by the Lucent Technologies, USA, https://www.r-project.org) and RStudio basement (version 1.4.1717, RStudio, USA; https://www.rstudio.com/products/rstudio/). For continuous variables, the independent Student’s t-test was used if the variables met the Gaussian distribution; if not, the Wilcoxon test was used. The Chi-squared test was used for categorical variables, and the Wilcoxon test was used for signed-rank variables. Correlation analysis was performed using Pearson’s or Spearman’s coefficient. A two-sided *p* value < 0.05 was considered to indicate statistical significance.

## 5. Conclusions

In conclusion, we successfully established a DILI diagnostic prediction model based on the total weight method by identifying several DILI microarrays in the GEO database and using six ML algorithms. A series of analyses and confirmations revealed that DDIT3, GADD45A, and RBM24 may help identify patients with DILI and become useful biomarkers for the clinical diagnosis of DILI.

## Figures and Tables

**Table 1:** The error rate of the six MLs.

MLs	Training Set (%)	Testing Set (%)
Lasso	5.00	11.10
SVM	3.80	7.40
RF	1.90	0.00
GBM	14.00	14.80
NN	5.00	11.10
DT	9.50	14.80

**Table 2:** The summation of the normalized weights of the six MLs.

Genes	Lasso	SVM	RF	NN	GBM	DT	Total Weights
DDIT3	0.914222	1	1	0.220019	1	1	5.134241
GADD45A	1	0.701511	0.595707	0.857692	0.138689	0.292928	3.586528
RBM24	0.032375	0.891609	0.762935	1	0.616145	0.217391	3.520456
SLC3A2	0.846442	0.62703	0.562559	0.690844	0.241597	0.432583	3.401055
IFRD1	0	0.542098	0.524983	0.365069	0.038438	0.26087	1.731457
GDF15	0	0.3516	0.383822	0.786062	0.00509	0.032059	1.558633
JMY	0	0.353108	0.433204	0.305061	0.123692	0.26087	1.475935
UPP1	0	0.512905	0.463177	0.396437	0.053665	0.032059	1.458243
CPEB4	0	0.165532	0.26272	0.743079	0.004831	0	1.176162
PPP1R15A	0	0.079463	0.222258	0.823152	0.006435	0	1.131307
PIM1	0	0.15555	0.307897	0.231436	0.113818	0.224411	1.033112
LDLR	0	0.055148	0.191678	0.572498	0.006348	0.064117	0.889789
MAFF	0	0.124409	0.208423	0.449151	0.030866	0	0.812849
TRIB3	0	0.180949	0.24403	0.349341	0.019652	0	0.793972
ASNS	0	0.25119	0.29182	0.183775	0.02987	0	0.756656
SLC7A11	0	0.141399	0.208826	0.348854	0.008297	0	0.707376
SLC20A1	0	0.308733	0.34717	0.017776	0.029455	0	0.703135
MTHFD2	0	0.079061	0.176848	0.379338	0.005218	0	0.640465
GPCPD1	0	0.204886	0.331006	0.017325	0.046414	0	0.599631
NR1D2	0	0.233607	0.284661	0.038773	0.021109	0	0.57815
GADD45B	0	0.124042	0.235818	0.142752	0.027562	0	0.530174

## Data Availability

The data sets analyzed in this study are available in the GEO database. All of the multiple microarrays, including GSE48634, GSE6731, GSE114527, GSE13367, GSE36807, GSE3629, GSE53306, GSE87473, GSE74265, and GSE96665, are derived from this database. The original contributions presented in this study are included in the manuscript/supplementary materials. For further enquiries, please contact the corresponding author.

## Supplementary Materials

The following supporting information can be downloaded at: https://www.mdpi.com/article/10.3390/ijms231911945/s1, The following supporting information include: Figure S1: The correlation map between immune cells in the control and DILI individuals. Figure S2: The linear regression maps of these four genes correlated with their respective significant immune cells. Table S1: The information of the 132 data that were selected from the six data sets. Table S2: The 21 differently expressed genes in the control and DILI samples. Table S3: The details of 94 GO enrichment. Table S4: The details of 18 KEGG enrichment. Table S5: The details of 19 GSEA enrichment. Table S6: The primary weight in the six ML algorithms.
