# Supplementary material for: Identification of Drug-Induced Liver Injury Biomarkers from Multiple Microarrays Based on Machine Learning and Bioinformatics Analysis
Source: Int J Mol Sci. 2022 Oct 8;23(19):11945. doi: 10.3390/ijms231911945 (PMC9570393; doi:10.3390/ijms231911945)
Supplement: Supplementary file 1 [file ijms-23-11945-s001.zip › ijms-1899103-supplementary.pdf]

Identification of drug-induced liver injury biomarkers from multiple microarrays based on machine learning and bioinformatics analysis

Content

Table S1 The information of 132 data which were selected from the 6 data sets.

Table S2 The 21 DEGs in control and DILI samples.

Table S3 The details of 94 GO enrichment.

Table S4 The details of 18 KEGG enrichment.

Table S5 The details of 19 GSEA enrichment.

Table S6 The primary weight in the six machine learning algorithms.

Figure. S1 The correlation map between immune cells in control and DILI individuals.

Figure. S2 The linear regression maps of these four genes correlated with their respective significant immune cells.

Table S1 The information of 132 data which were selected from the 6 datasets.

group	series name	GEO accession	type	source_name_ch1	organism_ch1
Con	GSE93840	GSM2463738	RNA	Primary human hepatocyte 3D spheroid	Homo sapiens
		GSM2463739	RNA	Primary human hepatocyte 3D spheroid	Homo sapiens
		GSM2463740	RNA	Primary human hepatocyte 3D spheroid	Homo sapiens
		GSM2463726	RNA	Primary human hepatocyte 3D spheroid	Homo sapiens
		GSM2463727	RNA	Primary human hepatocyte 3D spheroid	Homo sapiens
		GSM2463728	RNA	Primary human hepatocyte 3D spheroid	Homo sapiens
	GSE54254	GSM1311168	RNA	HepG2 ATCC, Wesel, Germany	Homo sapiens
		GSM1311142	RNA	HepG2 ATCC, Wesel, Germany	Homo sapiens
		GSM1311116	RNA	HepG2 ATCC, Wesel, Germany	Homo sapiens
		GSM1311090	RNA	HepG2 ATCC, Wesel, Germany	Homo sapiens
	GSE54255	GSM1311186	RNA	human liver	Homo sapiens
		GSM1311188	RNA	human liver	Homo sapiens
		GSM1311190	RNA	human liver	Homo sapiens
		GSM1311192	RNA	human liver	Homo sapiens
		GSM1311194	RNA	human liver	Homo sapiens
	GSE147866	GSM4447381	RNA	PBMCs, with HepaRG, DMSO, 24 hours	Homo sapiens
		GSM4447370	RNA	PBMCs, with HepG2, DMSO, 24 hours	Homo sapiens
	GSE102006	GSM2720396	RNA	HepG2	Homo sapiens
		GSM2720550	RNA	HepG2	Homo sapiens
		GSM2720554	RNA	HepG2	Homo sapiens

		GSM2720398	RNA	HepG2	Homo sapiens
		GSM2720552	RNA	HepG2	Homo sapiens
		GSM2720546	RNA	HepG2	Homo sapiens
	GSE99878	GSM2656616	RNA	13_1_72_Vehicle_0 (name_donor_time_treatment_dose)	Homo sapiens
		GSM2656617	RNA	34_2_72_Vehicle_0 (name_donor_time_treatment_dose)	Homo sapiens
		GSM2656618	RNA	55_3_72_Vehicle_0 (name_donor_time_treatment_dose)	Homo sapiens
		GSM2656613	RNA	7_1_24_Vehicle_0 (name_donor_time_treatment_dose)	Homo sapiens
		GSM2656614	RNA	28_2_24_Vehicle_0 (name_donor_time_treatment_dose)	Homo sapiens
		GSM2656615	RNA	49_3_24_Vehicle_0 (name_donor_time_treatment_dose)	Homo sapiens
DILI	GSE93840	GSM2463744	RNA	Primary human hepatocyte 3D spheroid	Homo sapiens
		GSM2463745	RNA	Primary human hepatocyte 3D spheroid	Homo sapiens
		GSM2463746	RNA	Primary human hepatocyte 3D spheroid	Homo sapiens
		GSM2463741	RNA	Primary human hepatocyte 3D spheroid	Homo sapiens
		GSM2463742	RNA	Primary human hepatocyte 3D spheroid	Homo sapiens
		GSM2463743	RNA	Primary human hepatocyte 3D spheroid	Homo sapiens
		GSM2463747	RNA	Primary human hepatocyte 3D spheroid	Homo sapiens
		GSM2463748	RNA	Primary human hepatocyte 3D spheroid	Homo sapiens
		GSM2463749	RNA	Primary human hepatocyte 3D spheroid	Homo sapiens
		GSM2463732	RNA	Primary human hepatocyte 3D spheroid	Homo sapiens
		GSM2463733	RNA	Primary human hepatocyte 3D spheroid	Homo sapiens
		GSM2463734	RNA	Primary human hepatocyte 3D spheroid	Homo sapiens
		GSM2463729	RNA	Primary human hepatocyte 3D spheroid	Homo sapiens

	GSM2463730	RNA	Primary human hepatocyte 3D spheroid	Homo sapiens
	GSM2463731	RNA	Primary human hepatocyte 3D spheroid	Homo sapiens
	GSM2463735	RNA	Primary human hepatocyte 3D spheroid	Homo sapiens
	GSM2463736	RNA	Primary human hepatocyte 3D spheroid	Homo sapiens
	GSM2463737	RNA	Primary human hepatocyte 3D spheroid	Homo sapiens
GSE54254	GSM1311181	RNA	HepG2 ATCC, Wesel, Germany	Homo sapiens
	GSM1311155	RNA	HepG2 ATCC, Wesel, Germany	Homo sapiens
	GSM1311129	RNA	HepG2 ATCC, Wesel, Germany	Homo sapiens
	GSM1311103	RNA	HepG2 ATCC, Wesel, Germany	Homo sapiens
	GSM1311171	RNA	HepG2 ATCC, Wesel, Germany	Homo sapiens
	GSM1311145	RNA	HepG2 ATCC, Wesel, Germany	Homo sapiens
	GSM1311119	RNA	HepG2 ATCC, Wesel, Germany	Homo sapiens
	GSM1311093	RNA	HepG2 ATCC, Wesel, Germany	Homo sapiens
	GSM1311176	RNA	HepG2 ATCC, Wesel, Germany	Homo sapiens
	GSM1311150	RNA	HepG2 ATCC, Wesel, Germany	Homo sapiens
	GSM1311124	RNA	HepG2 ATCC, Wesel, Germany	Homo sapiens
	GSM1311098	RNA	HepG2 ATCC, Wesel, Germany	Homo sapiens
GSE54255	GSM1311187	RNA	human liver	Homo sapiens
	GSM1311189	RNA	human liver	Homo sapiens
	GSM1311191	RNA	human liver	Homo sapiens
	GSM1311193	RNA	human liver	Homo sapiens
	GSM1311195	RNA	human liver	Homo sapiens

GSE147866	GSM4447382	RNA	PBMCs, with HepaRG, Amodiaquine, 24 hours	Homo sapiens
	GSM4447383	RNA	PBMCs, with HepaRG, Chloroquine, 24 hours	Homo sapiens
	GSM4447388	RNA	PBMCs, with HepaRG, Diclofenac, 24 hours	Homo sapiens
	GSM4447387	RNA	PBMCs, with HepaRG, EthacrinicAcid, 24 hours	Homo sapiens
	GSM4447384	RNA	PBMCs, with HepaRG, Ketoconazole, 24 hours	Homo sapiens
	GSM4447389	RNA	PBMCs, with HepaRG, Ketorolac, 24 hours	Homo sapiens
	GSM4447391	RNA	PBMCs, with HepaRG, Levofloxacin, 24 hours	Homo sapiens
	GSM4447385	RNA	PBMCs, with HepaRG, Mebendazole, 24 hours	Homo sapiens
	GSM4447386	RNA	PBMCs, with HepaRG, TienilicAcid, 24 hours	Homo sapiens
	GSM4447390	RNA	PBMCs, with HepaRG, Trovafloxacin, 24 hours	Homo sapiens
	GSM4447371	RNA	PBMCs, with HepG2, Amodiaquine, 24 hours	Homo sapiens
	GSM4447372	RNA	PBMCs, with HepG2, Chloroquine, 24 hours	Homo sapiens
	GSM4447377	RNA	PBMCs, with HepG2, Diclofenac, 24 hours	Homo sapiens
	GSM4447376	RNA	PBMCs, with HepG2, EthacrinicAcid, 24 hours	Homo sapiens
	GSM4447373	RNA	PBMCs, with HepG2, Ketoconazole, 24 hours	Homo sapiens
	GSM4447378	RNA	PBMCs, with HepG2, Ketorolac, 24 hours	Homo sapiens
	GSM4447380	RNA	PBMCs, with HepG2, Levofloxacin, 24 hours	Homo sapiens
	GSM4447374	RNA	PBMCs, with HepG2, Mebendazole, 24 hours	Homo sapiens
	GSM4447375	RNA	PBMCs, with HepG2, TienilicAcid, 24 hours	Homo sapiens
	GSM4447379	RNA	PBMCs, with HepG2, Trovafloxacin, 24 hours	Homo sapiens
GSE102006	GSM2720424	RNA	HepG2	Homo sapiens
	GSM2720472	RNA	HepG2	Homo sapiens

GSM2720520	RNA	HepG2	Homo sapiens
GSM2720432	RNA	HepG2	Homo sapiens
GSM2720480	RNA	HepG2	Homo sapiens
GSM2720528	RNA	HepG2	Homo sapiens
GSM2720572	RNA	HepG2	Homo sapiens
GSM2720604	RNA	HepG2	Homo sapiens
GSM2720632	RNA	HepG2	Homo sapiens
GSM2720428	RNA	HepG2	Homo sapiens
GSM2720476	RNA	HepG2	Homo sapiens
GSM2720524	RNA	HepG2	Homo sapiens
GSM2720420	RNA	HepG2	Homo sapiens
GSM2720468	RNA	HepG2	Homo sapiens
GSM2720516	RNA	HepG2	Homo sapiens
GSM2720568	RNA	HepG2	Homo sapiens
GSM2720600	RNA	HepG2	Homo sapiens
GSM2720628	RNA	HepG2	Homo sapiens
GSM2720440	RNA	HepG2	Homo sapiens
GSM2720488	RNA	HepG2	Homo sapiens
GSM2720536	RNA	HepG2	Homo sapiens
GSM2720448	RNA	HepG2	Homo sapiens
GSM2720496	RNA	HepG2	Homo sapiens
GSM2720544	RNA	HepG2	Homo sapiens

	GSM2720582	RNA	HepG2	Homo sapiens
	GSM2720614	RNA	HepG2	Homo sapiens
	GSM2720642	RNA	HepG2	Homo sapiens
	GSM2720444	RNA	HepG2	Homo sapiens
	GSM2720492	RNA	HepG2	Homo sapiens
	GSM2720540	RNA	HepG2	Homo sapiens
	GSM2720436	RNA	HepG2	Homo sapiens
	GSM2720484	RNA	HepG2	Homo sapiens
	GSM2720532	RNA	HepG2	Homo sapiens
	GSM2720578	RNA	HepG2	Homo sapiens
	GSM2720610	RNA	HepG2	Homo sapiens
	GSM2720638	RNA	HepG2	Homo sapiens
GSE99878	GSM2656652	RNA	17_1_72_Tolvaptan_20 (name_donor_time_treatment_dose)	Homo sapiens
	GSM2656653	RNA	38_2_72_Tolvaptan_20 (name_donor_time_treatment_dose)	Homo sapiens
	GSM2656654	RNA	59_3_72_Tolvaptan_20 (name_donor_time_treatment_dose)	Homo sapiens
	GSM2656661	RNA	18_1_72_Tolvaptan_50 (name_donor_time_treatment_dose)	Homo sapiens
	GSM2656662	RNA	39_2_72_Tolvaptan_50 (name_donor_time_treatment_dose)	Homo sapiens
	GSM2656663	RNA	60_3_72_Tolvaptan_50 (name_donor_time_treatment_dose)	Homo sapiens
	GSM2656649	RNA	11_1_24_Tolvaptan_20 (name_donor_time_treatment_dose)	Homo sapiens
	GSM2656650	RNA	32_2_24_Tolvaptan_20 (name_donor_time_treatment_dose)	Homo sapiens
	GSM2656651	RNA	53_3_24_Tolvaptan_20 (name_donor_time_treatment_dose)	Homo sapiens
	GSM2656658	RNA	12_1_24_Tolvaptan_50 (name_donor_time_treatment_dose)	Homo sapiens

GSM2656659	RNA	33_2_24_Tolvaptan_50 (name_donor_time_treatment_dose)	Homo sapiens
GSM2656660	RNA	54_3_24_Tolvaptan_50 (name_donor_time_treatment_dose)	Homo sapiens

Table S2 The 21 differently expressed genes in control and DILI samples.

ID	logFC	AveExpr	t	P.Value	adj.P.Val	B
DDIT3	1.392995	8.108105	4.877077	3.74×10^{-6}	0.007977	4.10495
GADD45A	1.040195	10.98057	4.837684	4.40×10^{-6}	0.007977	3.95909
SLC3A2	0.853186	9.896595	4.794309	5.27×10^{-6}	0.007977	3.799329
UPP1	0.902094	7.907806	4.313963	3.57×10^{-5}	0.016524	2.092419
IFRD1	0.861211	9.161969	4.259608	4.40×10^{-5}	0.016524	1.906863
GDF15	1.231028	13.31132	4.255195	4.47×10^{-5}	0.016524	1.89187
JMY	0.834875	7.332827	4.21754	5.16×10^{-5}	0.016524	1.764369
SLC20A1	0.886059	11.71792	4.182897	5.89×10^{-5}	0.016524	1.64776
TRIB3	0.94512	11.6285	4.172851	6.12×10^{-5}	0.016524	1.614072
NR1D2	1.016956	7.182853	4.121886	7.41×10^{-5}	0.017233	1.444041
CPEB4	0.892595	8.615527	3.965255	0.000132	0.020825	0.930829
GPCPD1	1.124995	8.289366	3.832121	0.000214	0.023106	0.505993
RBM24	1.192887	5.726108	3.71446	0.000325	0.0269	0.139526
MTHFD2	0.869899	9.147679	3.677651	0.000369	0.0269	0.026649
ASNS	0.901988	10.96432	3.649494	0.000407	0.0269	-0.05912
MAFF	1.029844	8.130305	3.636508	0.000426	0.0269	-0.0985
SLC7A11	1.063315	5.9342	3.468887	0.000753	0.035522	-0.59713
LDLR	0.899772	9.550464	3.466114	0.00076	0.035522	-0.60523
PIM1	1.05971	8.72418	3.392995	0.000968	0.03893	-0.81683
GADD45B	1.02509	8.819941	3.383714	0.000998	0.03893	-0.84343
PPP1R15A	1.032055	7.02125	3.305436	0.001289	0.043564	-1.0655

logFC, log fold change; AveExpr, Average expression value; adj.P.Val, Adjusted p-values; B is the logarithmic value of the standard deviation obtained by empirical Bayes.

Table S3 The details of 94 GO enrichment.

ONTOLOGY	ID	Description	pvalue	qvalue	geneID	Count
BP	GO:0031667	response to nutrient levels	1.46×10^{-8}	$6.27\times^{10}$-6	DDIT3/UPP1/GDF15/JMY/CPEB4/ASNS/LDLR/PIM1	8
BP	GO:0009991	response to extracellular stimulus	2.55×10^{-8}	6.27×10^{-6}	DDIT3/UPP1/GDF15/JMY/CPEB4/ASNS/LDLR/PIM1	8
BP	GO:0071496	cellular response to external stimulus	8.28×10^{-7}	0.000135945	GADD45A/UPP1/JMY/CPEB4/ASNS/PIM1	6
BP	GO:0042594	response to starvation	2.08×10^{-6}	0.000256089	DDIT3/UPP1/JMY/CPEB4/ASNS	5
BP	GO:0031669	cellular response to nutrient levels	3.13×10^{-6}	0.000308055	UPP1/JMY/CPEB4/ASNS/PIM1	5
BP	GO:0031668	cellular response to extracellular stimulus	6.19×10^{-6}	0.000508348	UPP1/JMY/CPEB4/ASNS/PIM1	5
BP	GO:0042149	cellular response to glucose starvation	2.28×10^{-5}	0.001468503	UPP1/CPEB4/ASNS	3
BP	GO:0007519	skeletal muscle tissue development	2.38×10^{-5}	0.001468503	NR1D2/GPCPD1/RBM24/MAFF	4
BP	GO:0009267	cellular response to starvation	2.70×10^{-5}	0.001478543	UPP1/JMY/CPEB4/ASNS	4

BP	GO:0060538	skeletal muscle organ development	3.19×10^{-5}	0.0015738	NR1D2/GPCPD1/RBM24/MAFF	4
BP	GO:0070059	intrinsic apoptotic signaling pathway in response to endoplasmic reticulum stress	4.57×10^{-5}	0.002046072	DDIT3/TRIB3/PPP1R15A	3
BP	GO:0035914	skeletal muscle cell differentiation	5.02×10^{-5}	0.002060062	NR1D2/RBM24/MAFF	3
BP	GO:0060537	muscle tissue development	6.93×10^{-5}	0.002625332	NR1D2/GPCPD1/RBM24/MAFF/PIM1	5
BP	GO:0045445	myoblast differentiation	9.69×10^{-5}	0.003409662	DDIT3/IFRD1/RBM24	3
BP	GO:0036499	PERK-mediated unfolded protein response	0.000179873	0.005907406	DDIT3/PPP1R15A	2
BP	GO:2001014	regulation of skeletal muscle cell differentiation	0.000223071	0.006868237	NR1D2/RBM24	2
BP	GO:0140467	integrated stress response signaling	0.000270842	0.007848555	DDIT3/PPP1R15A	2
BP	GO:0097193	intrinsic apoptotic signaling pathway	0.000287963	0.007881099	DDIT3/JMY/TRIB3/PPP1R15A	4
BP	GO:2000765	regulation of cytoplasmic translation	0.000441409	0.011356836	CPEB4/RBM24	2

BP	GO:0007517	muscle organ development	0.000461068	0.011356836	NR1D2/GPCPD1/RBM24/MAFF	4
BP	GO:0045598	regulation of fat cell differentiation	0.000507843	0.011913318	DDIT3/TRIB3/PIM1	3
BP	GO:1900745	positive regulation of p38MAPK cascade	0.000577646	0.012934851	GADD45A/GADD45B	2
BP	GO:0046475	glycerophospholipid catabolic process	0.000652467	0.013966427	GPCPD1/LDLR	2
BP	GO:0046434	organophosphate catabolic process	0.000680416	0.013966427	UPP1/GPCPD1/LDLR	3
BP	GO:0006984	ER-nucleus signaling pathway	0.00114292	0.02165533	DDIT3/PPP1R15A	2
BP	GO:0089718	amino acid import across plasma membrane	0.00114292	0.02165533	SLC3A2/SLC7A11	2
BP	GO:1900744	regulation of p38MAPK cascade	0.001194068	0.021786496	GADD45A/GADD45B	2
BP	GO:0045936	negative regulation of phosphate metabolic process	0.001291216	0.022118932	GADD45A/TRIB3/GADD45B/PPP1R15A	4
BP	GO:0010563	negative regulation of phosphorus metabolic process	0.001302087	0.022118932	GADD45A/TRIB3/GADD45B/PPP1R15A	4
BP	GO:0009395	phospholipid catabolic process	0.001523629	0.024513176	GPCPD1/LDLR	2
BP	GO:0043090	amino acid import	0.001582315	0.024513176	SLC3A2/SLC7A11	2

BP	GO:0006469	negative regulation of protein kinase activity	0.001640454	0.024513176	GADD45A/TRIB3/GADD45B	3
BP	GO:0045661	regulation of myoblast differentiation	0.001642068	0.024513176	DDIT3/RBM24	2
BP	GO:0038066	p38MAPK cascade	0.001827715	0.026482058	GADD45A/GADD45B	2
BP	GO:0045599	negative regulation of fat cell differentiation	0.001956778	0.027542015	DDIT3/TRIB3	2
BP	GO:0033673	negative regulation of kinase activity	0.002197842	0.02961679	GADD45A/TRIB3/GADD45B	3
BP	GO:0045444	fat cell differentiation	0.002224423	0.02961679	DDIT3/TRIB3/PIM1	3
BP	GO:0098656	anion transmembrane transport	0.002501338	0.031768913	SLC3A2/SLC20A1/SLC7A11	3
BP	GO:1902475	L-alpha-amino acid transmembrane transport	0.002515039	0.031768913	SLC3A2/SLC7A11	2
BP	GO:0046503	glycerolipid catabolic process	0.002589511	0.031891876	GPCPD1/LDLR	2
BP	GO:0015807	L-amino acid transport	0.002665019	0.032021276	SLC3A2/SLC7A11	2
BP	GO:0034976	response to endoplasmic reticulum stress	0.002798807	0.032828108	DDIT3/TRIB3/PPP1R15A	3
BP	GO:0051348	negative regulation of transferase activity	0.00321708	0.036078126	GADD45A/TRIB3/GADD45B	3

BP	GO:0030968	endoplasmic reticulum unfolded protein response	0.003222363	0.036078126	DDIT3/PPP1R15A	2
BP	GO:0009064	glutamine family amino acid metabolic process	0.003390794	0.037120268	ASNS/SLC7A11	2
BP	GO:1905897	regulation of response to endoplasmic reticulum stress	0.003739797	0.040050919	DDIT3/PPP1R15A	2
MF	GO:0015175	neutral amino acid transmembrane transporter activity	0.000896349	0.040547743	SLC3A2/SLC7A11	2
MF	GO:0015179	L-amino acid transmembrane transporter activity	0.00197452	0.040547743	SLC3A2/SLC7A11	2
MF	GO:0015291	secondary active transmembrane transporter activity	0.00211696	0.040547743	SLC3A2/SLC20A1/SLC7A11	3
MF	GO:0022853	active ion transmembrane transporter activity	0.002486983	0.040547743	SLC3A2/SLC20A1/SLC7A11	3
MF	GO:0015171	amino acid transmembrane transporter activity	0.003705469	0.040547743	SLC3A2/SLC7A11	2
MF	GO:0015297	antiporter activity	0.004149038	0.040547743	SLC3A2/SLC7A11	2

MF	GO:0022804	active transmembrane transporter activity	0.009060771	0.040547743	SLC3A2/SLC20A1/SLC7A11	3
MF	GO:0005432	calcium:sodium antiporter activity	0.010813336	0.040547743	SLC3A2	1
MF	GO:0015173	aromatic amino acid transmembrane transporter activity	0.010813336	0.040547743	SLC3A2	1
MF	GO:0016884	carbon-nitrogen ligase activity, with glutamine as amido-N-donor	0.010813336	0.040547743	ASNS	1
MF	GO:0015081	sodium ion transmembrane transporter activity	0.011380519	0.040547743	SLC3A2/SLC20A1	2
MF	GO:0043021	ribonucleoprotein complex binding	0.011380519	0.040547743	CPEB4/PIM1	2
MF	GO:0005436	sodium:phosphate symporter activity	0.011888539	0.040547743	SLC20A1	1
MF	GO:0015368	calcium:cation antiporter activity	0.011888539	0.040547743	SLC3A2	1
MF	GO:0043522	leucine zipper domain binding	0.011888539	0.040547743	DDIT3	1

MF	GO:0022858	alanine transmembrane transporter activity	0.012962632	0.040547743	SLC3A2	1
MF	GO:0042301	phosphate ion binding	0.012962632	0.040547743	MTHFD2	1
MF	GO:0097157	pre-mRNA intronic binding	0.012962632	0.040547743	RBM24	1
MF	GO:0046943	carboxylic acid transmembrane transporter activity	0.013488921	0.040547743	SLC3A2/SLC7A11	2
MF	GO:0005342	organic acid transmembrane transporter activity	0.01364559	0.040547743	SLC3A2/SLC7A11	2
MF	GO:0005313	L-glutamate transmembrane transporter activity	0.01510749	0.040547743	SLC7A11	1
MF	GO:0070700	BMP receptor binding	0.01510749	0.040547743	GDF15	1
MF	GO:1990825	sequence-specific mRNA binding	0.01510749	0.040547743	RBM24	1
MF	GO:0005041	low-density lipoprotein particle receptor activity	0.016178259	0.040547743	LDLR	1
MF	GO:0071933	Arp2/3 complex binding	0.016178259	0.040547743	JMY	1
MF	GO:0008514	organic anion transmembrane transporter activity	0.017117721	0.040547743	SLC3A2/SLC7A11	2

MF	GO:0000900	mRNA regulatory element binding translation repressor activity	0.017247921	0.040547743	CPEB4	1
MF	GO:0015172	acidic amino acid transmembrane transporter activity	0.017247921	0.040547743	SLC7A11	1
MF	GO:0030275	LRR domain binding	0.017247921	0.040547743	DDIT3	1
MF	GO:0072542	protein phosphatase activator activity	0.017247921	0.040547743	PPP1R15A	1
MF	GO:0043024	ribosomal small subunit binding	0.018316478	0.041426584	PIM1	1
MF	GO:0030169	low-density lipoprotein particle binding	0.019383931	0.041426584	LDLR	1
MF	GO:0030228	lipoprotein particle receptor activity	0.019383931	0.041426584	LDLR	1
MF	GO:0016646	oxidoreductase activity, acting on the CH-NH group of donors, NAD or NADP as acceptor	0.020450282	0.04242009	MTHFD2	1
MF	GO:0055106	ubiquitin-protein transferase regulator activity	0.022579679	0.044235044	TRIB3	1

MF	GO:0140416	transcription regulator inhibitor activity	0.022579679	0.044235044	DDIT3	1
MF	GO:0072349	modified amino acid transmembrane transporter activity	0.023642728	0.044255516	SLC7A11	1
MF	GO:0003730	mRNA 3'-UTR binding	0.023845136	0.044255516	CPEB4/RBM24	2
MF	GO:0019211	phosphatase activator activity	0.024704679	0.044675128	PPP1R15A	1
MF	GO:0030247	polysaccharide binding	0.026825289	0.046143629	GPCPD1	1
MF	GO:0070696	transmembrane receptor protein serine/threonine kinase binding	0.026825289	0.046143629	GDF15	1
MF	GO:0035925	mRNA 3'-UTR AU-rich region binding	0.02788395	0.046822674	RBM24	1
MF	GO:0015491	cation:cation antiporter activity	0.028941518	0.04746834	SLC3A2	1
MF	GO:0016645	oxidoreductase activity, acting on the CH-NH group of donors	0.031053375	0.048668447	MTHFD2	1
MF	GO:0033612	receptor serine/threonine kinase binding	0.031053375	0.048668447	GDF15	1
MF	GO:0008157	protein phosphatase 1 binding	0.032107666	0.048723205	PPP1R15A	1
MF	GO:0071813	lipoprotein particle binding	0.033160868	0.048723205	LDLR	1

MF	GO:0071814	protein-lipid complex binding	0.033160868	0.048723205	LDLR	1

BP: biological processes; MF, molecular functions.

Table S4 The details of 18 KEGG enrichment.

ID	Description	pvalue	qvalue	GeneID	Count
hsa04210	Apoptosis	0.001779	0.025515	DDIT3/GADD45A/GADD45B	3
hsa05216	Thyroid cancer	0.002024	0.025515	GADD45A/GADD45B	2
hsa04216	Ferroptosis	0.002482	0.025515	SLC3A2/SLC7A11	2
hsa05202	Transcriptional misregulation in cancer	0.004805	0.025515	DDIT3/GADD45A/GADD45B	3
hsa05213	Endometrial cancer	0.004913	0.025515	GADD45A/GADD45B	2
hsa05217	Basal cell carcinoma	0.005774	0.025515	GADD45A/GADD45B	2
hsa05218	Melanoma	0.007485	0.025515	GADD45A/GADD45B	2
hsa05223	Non-small cell lung cancer	0.007485	0.025515	GADD45A/GADD45B	2
hsa04115	p53 signaling pathway	0.007688	0.025515	GADD45A/GADD45B	2
hsa05214	Glioma	0.0081	0.025515	GADD45A/GADD45B	2
hsa05212	Pancreatic cancer	0.008311	0.025515	GADD45A/GADD45B	2
hsa05220	Chronic myeloid leukemia	0.008311	0.025515	GADD45A/GADD45B	2
hsa05210	Colorectal cancer	0.010546	0.029886	GADD45A/GADD45B	2

hsa05222	Small cell lung cancer	0.012001	0.031582	GADD45A/GADD45B	2
hsa04064	NF-kappa B signaling pathway	0.015162	0.035155	GADD45A/GADD45B	2
hsa04010	MAPK signaling pathway	0.015267	0.035155	DDIT3/GADD45A/GADD45B	3
hsa04110	Cell cycle	0.02178	0.047201	GADD45A/GADD45B	2
hsa04068	FoxO signaling pathway	0.023426	0.047948	GADD45A/GADD45B	2

GeneRatio BgRatio pvalue p.adjust qvalue

Table S5 The details of 19 GSEA enrichment.

ID	Enrichment Score	NES	pvalue	p.adjust	qvalues	rank	leading_edge
DNA_REPLICATION	-0.77288	-2.50492	1.86×10^{-9}	3.17×10^{-7}	2.40×10^{-7}	1258	tags=66%, list=14%, signal=56%
TGF_BETA_SIGNALING_PATHWAY	0.573032	2.076436	1.14×10^{-5}	0.000975	0.000738	2250	tags=57%, list=25%, signal=43%
MISMATCH_REPAIR	-0.73963	-2.15959	2.78×10^{-5}	0.001295	0.000981	932	tags=61%, list=10%, signal=55%
MAPK_SIGNALING_PATHWAY	0.408231	1.765777	3.03×10^{-5}	0.001295	0.000981	2162	tags=35%, list=24%, signal=27%

PROTEASOME	-0.56582	-1.96202	0.000109	0.003732	0.002825	2103	tags=41%, list=23%, signal=32%
ERBB_SIGNALING_PATHWAY	0.510567	1.931722	0.000232	0.006618	0.005011	1794	tags=43%, list=20%, signal=35%
PRIMARY_BILE_ACID_BIOSYNTHESIS	-0.78152	-2.02918	0.000281	0.006857	0.005192	687	tags=58%, list=8%, signal=54%
GLUTATHIONE_METABOLISM	-0.5292	-1.855	0.000368	0.007877	0.005964	1046	tags=42%, list=12%, signal=38%
PEROXISOME	-0.44482	-1.73201	0.000762	0.014477	0.010961	1900	tags=45%, list=21%, signal=36%
P53_SIGNALING_PATHWAY	0.507838	1.845689	0.001195	0.020443	0.015478	744	tags=24%, list=8%, signal=23%
BETA_ALANINE_METABOLISM	-0.65913	-1.8821	0.0017	0.025759	0.019503	1624	tags=82%, list=18%, signal=68%
GLYCOSYLPHOSPHATIDYLINOSITOL_GPI_ANCHOR_BIOSYNTHESIS	-0.59091	-1.81443	0.001808	0.025759	0.019503	2015	tags=57%, list=22%, signal=44%

PROPANOATE_METABOLISM	-0.55863	-1.78055	0.002063	0.02714	0.02055	2694	tags=59%, list=30%, signal=42%
NUCLEOTIDE_EXCISION_REPAIR	-0.48393	-1.69631	0.002477	0.028234	0.021378	996	tags=32%, list=11%, signal=29%
WNT_SIGNALING_PATHWAY	0.39428	1.580015	0.002333	0.028234	0.021378	2250	tags=39%, list=25%, signal=29%
PURINE_METABOLISM	-0.36366	-1.56048	0.003071	0.031095	0.023544	1945	tags=34%, list=22%, signal=27%
PYRIMIDINE_METABOLISM	-0.38255	-1.54577	0.003091	0.031095	0.023544	1708	tags=30%, list=19%, signal=24%
HISTIDINE_METABOLISM	-0.60132	-1.7902	0.003475	0.033011	0.024994	1738	tags=55%, list=19%, signal=44%
NEUROACTIVE_LIGAND_RECEPTOR_INTERACTION	0.49738	1.698239	0.00431	0.038794	0.029373	1772	tags=48%, list=20%, signal=38%

NES, normalized enrichment score; Rank represents the position of the corresponding gene in the ranked gene list L when the ES value is the largest; Leading edge, the leading gene, the gene member that contributes the most to the enrichment. Tags represent the percentage of core genes in the total

number of genes in the gene set. List represents the percentage of core genes over all genes, signal, and signal is the enriched signal strength calculated by combining the first two statistics together.

Table S6 The primary weight in the six machine learning algorithms.

Genes	Lasso	SVM	RF	NN	GBM	DT
GADD45A	0.389987	75.02976	2.491505	1.862589	21.14452	3.527813
DDIT3	0.356534	106.9544	4.182433	-0.4778	152.4595	12.04327
SLC3A2	0.330101	67.06369	2.352865	1.500257	36.83381	5.209707
RBM24	0.012626	95.36155	3.190923	2.171629	93.93723	2.618101
ASNS	0	26.86585	1.220518	-0.39909	4.554034	0
CPEB4	0	17.70437	1.098809	-1.61369	0.73651	0
GADD45B	0	13.26686	0.986291	-0.31	4.202093	0
GDF15	0	37.60517	1.60531	-1.70703	0.776083	0.386091
GPCPD1	0	21.91348	1.38441	-0.03762	7.076269	3.141722
IFRD1	0	57.97979	2.195707	0.792794	5.860219	3.141722
JMY	0	37.76645	1.811846	0.66248	18.85799	0
LDLR	0	5.898298	0.801679	-1.24325	0.967745	0.772183
MAFF	0	13.30606	0.871715	0.975389	4.705853	0
MTHFD2	0	8.455915	0.739653	-0.82378	0.795486	0

NR1D2	0	24.98527	1.190576	-0.0842	3.218286	0
PIM1	0	16.63679	1.287759	0.502593	17.35262	2.70264
PPP1R15A	0	8.4989	0.929577	-1.78758	0.981129	0
SLC20A1	0	33.02035	1.452016	0.038604	4.490701	0
SLC7A11	0	15.1232	0.873401	-0.75758	1.264896	0
TRIB3	0	19.35333	1.020637	0.75864	2.996177	0
UPP1	0	54.85751	1.937208	-0.86091	8.181687	0.386091

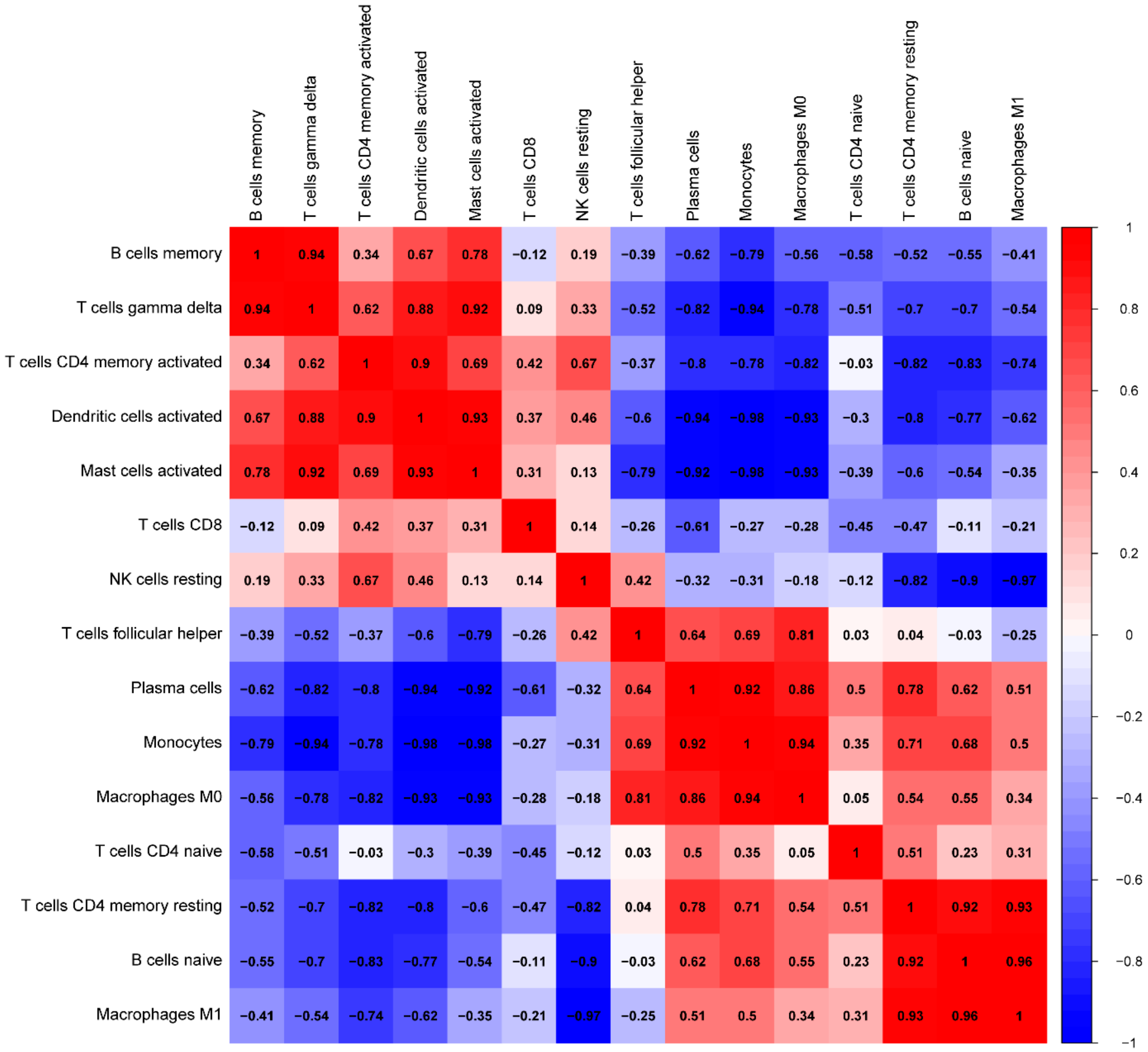

Figure S1 The correlation map between immune cells in control and DILI individuals.

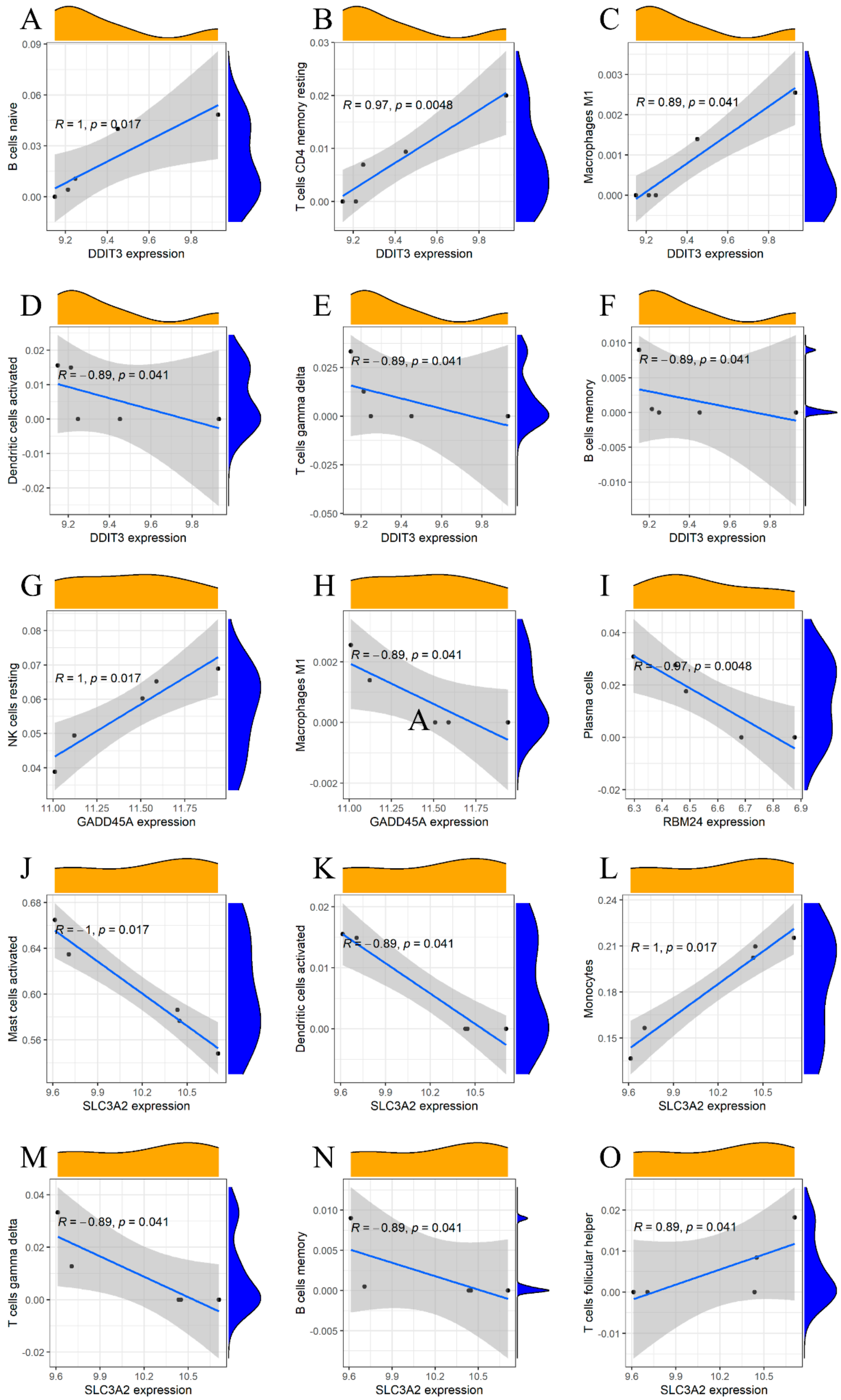
A
B cells naive
R = 1, p = 0.017
DDIT3 expression
B
T cells CD4 memory resting
R = 0.97, p = 0.0048
DDIT3 expression
C
Macrophages M1
R = 0.89, p = 0.041
DDIT3 expression
D
Dendritic cells activated
R = −0.89, p = 0.041
DDIT3 expression
E
T cells gamma delta
R = −0.89, p = 0.041
DDIT3 expression
F
B cells memory
R = −0.89, p = 0.041
DDIT3 expression
G
NK cells resting
R = 1, p = 0.017
GADD45A expression
H
Macrophages M1
R = −0.89, p = 0.041
A
GADD45A expression
I
Plasma cells
R = −0.97, p = 0.0048
RBM24 expression
J
Mast cells activated
R = −1, p = 0.017
SLC3A2 expression
K
Dendritic cells activated
R = −0.89, p = 0.041
SLC3A2 expression
L
Monocytes
R = 1, p = 0.017
SLC3A2 expression
M
T cells gamma delta
R = −0.89, p = 0.041
SLC3A2 expression
N
B cells memory
R = −0.89, p = 0.041
SLC3A2 expression
O
T cells follicular helper
R = 0.89, p = 0.041
SLC3A2 expression

Figure S2 The linear regression maps of these four genes correlated with their respective significant immune cells were shown in Fig. S2. A-F, The linear regression maps of DDIT3 associated with B cells naive, T cells CD4 memory resting, Macrophages M1, Dendritic cells activated, T cells gamma delta, B cells memory; G-H, The linear regression maps of GADD45A associated with NK cells resting and Macrophages M1; I, The linear regression maps of RBM24 associated with Plasma cells; J-O, The linear regression maps of SLC3A2 associated with Mast cells activated, Dendritic cells activated, Monocytes, T cells gamma delta, B cells memory, and T cells follicular helper.
